# Risk Factors Related to Esophageal Cancer, a Case-Control Study in Herat Province of Afghanistan

**DOI:** 10.34172/aim.2022.107

**Published:** 2022-10-01

**Authors:** Sultan Eser, Su Özgür, Nasar Ahmad Shayan, Mohammed Haris Abdianwall

**Affiliations:** ^1^Balıkesir University, Faculty of Medicine, Department of Public Health, 10145, Balıkesir, Turkey; ^2^Ege University Faculty of Medicine Department of Biostatistics and Medical Informatics, 35040 Bornova, İzmir, Turkey; ^3^Western University, Faculty of Medicine, Department of Epidemiology and Biostatistics, London- On, Canada; ^4^Nangarhar University, Faculty of Medicine, Department of Clinical Ophthalmology, Celalabad, Afghanistan

**Keywords:** Esophageal cancer, Esophageal squamous cell carcinoma, Herat region, Risk factors

## Abstract

**Background:**

The Herat province of Afghanistan is located on the Asian Esophageal Cancer Belt (AECB), a wide area in Central and Eastern Asia where very high rates of esophageal cancer (EC) have been observed. Several risk factors have been reported in the AECB Region by previous studies. Considering lack of information in Afghanistan on this issue, a study was conducted to determine the major risk factors related to EC in order to guide protective measures.

**Methods:**

A population-based case-control study was performed from July 2015 to August 2016 among 657 EC patients in the Herat Province and 180 histopathological confirmed cases and 189 controls were interviewed. A structured questionnaire was used and face-to-face interviews were conducted.

**Results:**

Low body mass index (BMI), low socio-economic status, family history of EC, consumption of dark tea, very hot beverage and qulurtoroosh were found to be statistically significant for EC and esophageal squamous cell carcinoma (ESCC) in univariate analyses. According to multivariate analyses, sex (OR=2.268; 95% CI=1.238–4.153), very hot beverages (OR=2.253; 95% CI=1.271– 3.996), qulurtoroosh (OR=5.679; 95% CI=1.787–18.815), dark tea (OR=2.757; 95% CI=1.531–4.967), high previous BMI (OR=0.215; 95% CI=0.117–0.431) and low socio-economic status (OR=1.783; 95% CI=1.007–3.177) were associated with ESCC. Being male was found to increase the risk of ESCC with OR=2.268 (95% CI=1.238–4.153).

**Conclusion:**

Consuming very hot beverages dark tea and a local food, qulurtoroosh, were found as important risk factors for EC. Our findings warrant further studies and necessitate the implementation of protective measures for EC which is one of the leading cancers in the region.

## Introduction

 Esophageal cancer (EC) is the 8^th^ most common cancer and 6^th^ most common cause of cancer death in the world. Worldwide incidence, prevalence and mortality were respectively estimated at 604 100 and 666 388 cases and 544 076 deaths in 2020.^1^

 Cancer of the esophagus is characterized by a specific geographical distribution in the world. Up to 20-fold higher risks for EC have been reported from several countries in Central and Eastern Asia, called the Asian Esophageal Cancer Belt (AECB), which stretches eastward from the Eastern part of Turkey and Iran through Turkmenistan, Northern Afghanistan, Uzbekistan and Kazakhstan into northern China and Mongolia.^2^ Cancer of the esophagus mainly consists of two types: esophageal squamous cell carcinoma (ESCC) and esophageal adenocarcinoma (EAC).^3^ ESCC is the most common histological type of EC in low- and middle-income countries and constitutes 90% of the cases in the AECB.^3,4^ In general, advanced age, male sex and black race have a higher chance of being diagnosed with EC in North America and Western Europe.^5^ Tobacco use, alcohol consumption and nutritional imbalance (low intake of micronutrients such as vitamin A, C, E, riboflavin, zinc, selenium and low intake of fresh fruits and vegetables) are the factors related to ESCC while Barrett’s esophagus, chronic gastro-esophageal reflux disease, overweight and obesity, smoking and nutritional imbalance (excessive carbohydrate intake & low fruit and vegetable intake) were found as risk factors for EAC.^6^

 In high-risk areas, the risk factors for ESCC are thought to be poor nutritional status, low intake of fruit and vegetable and drinking beverage in high temperature, whereas in Western countries, smoking and excessive alcohol consumption and low fruit and vegetable consumption account for about more than 90% of the total cases of ESCC.^7^

**Figure 1:**
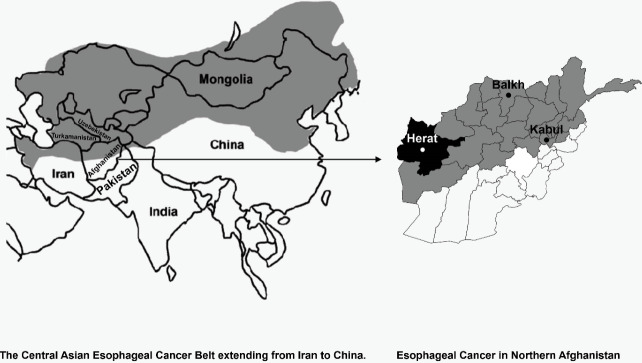
Location of Afghanistan in South-Central Asia^10^

 Afghanistan is located in South-Central Asia (Figure 1). Two of six regions (North-Eastern, Northern and Western region) are located on the AECB, with high incidence of EC. Hospital-based studies have reported high proportions of ECs among the patients admitted to the hospitals and despite the lack of population-based figures, EC is defined as one of the major cancers in the region.^8,9^

 There is lack of knowledge about the risk factors of esophageal carcinoma in regions with high incidence and prevalence of esophageal carcinoma in Afghanistan. It is necessary to identify the risk factors of EC for each geographical location to implement meaningful preventive strategies in order to control EC. Therefore, a case control study was designed to determine the major risk factors related to the cancer of esophagus in the region. The study was conducted in the four endoscopic examination centers (all gastroscopic examinations take place in these four centers) located in the Herat province, which is located in the Western region of Afghanistan and on the AECB. We aimed to determine the risk factors related to EC in the Herat province of Afghanistan.

## Materials and Methods

 We conducted a population-based case-control study from July 2015 to August 2016 in the central capital of the Herat Province. Adult men and women, who referred to all four endoscopic examination centers located in the capital of the Herat Province, diagnosed with EC by endoscopy and confirmed by histopathologic examination, who lived in the Herat Province and accepted our invitation were included in the study as cases. The criteria for participant eligibility were as follows: (1) age 18 and above; (2) voluntary participation (3) completing all phases of the examination, including endoscopy and pathology, and (4) no previous psychological disorders which affect the memory (e.g., Alzheimer’s disease).

 Totally, 657 patients were diagnosed as probable esophageal carcinoma in four endoscopic centers and 460 (70%) of them accepted biopsy for histopathologic examination. The reasons for rejecting biopsy and histopathologic examination were economic problems, fear, anxiety, distrust, and probability of bleeding risk during the biopsy procedure. On histopathologic examinations, out of 460 patients, 180 were confirmed as having esophageal carcinoma and the remaining had benign lesions of the esophagus. All 180 esophageal carcinoma cases along with 189 controls entered the study.

 The controls were selected as similarly as possible to the cases in terms of age and gender. Each of the patients were asked to invite his/her neighbor/friend/family similar to him/her with reference to age and gender as control subjects. Some controls were selected from the dermatology outpatient departments, when no response was obtained from the neighbor/friend/family of the cases.

 Face-to-face interviews were conducted for cases in the histopathology unit, immediately after the confirmation of EC. For controls, face-to-face interviews were also conducted in the community and/or in the hospital by trained interviewers.

 The minimum sample size for each group (case and control) was calculated as 174 considering OR = 3 and 95% confidence level with 80% desired power.

 A structured questionnaire, developed by the research team, was used for data collection. It includes information about the demographic and lifestyle characteristics during most of their life before they became cases for the case subjects and before the interview for control subjects. The questionnaire form consisted of three parts. In the first part, socio-demographic characteristics such as age, sex, literacy, marital status, nationality, place of residence, self-reported economic status (SES) and working status for most of his/her life were recorded. In the second part, questions related to behavior and lifestyle such as tobacco smoking, alcohol consumption, dietary intake (type and frequency) and body mass index (BMI) status were asked. In the third part, history of some chronic diseases such as gastroesophageal reflux, history of EC among relatives and presence of *Helicobacter pylori* were questioned. Also, since weight loss is a prognostic factor for EC, BMI was asked in two questions as previous weight (one/two years ago) and current weight to be able to avoid the recall bias and previous BMI was included in the analysis. Data about the disease (EC), such as diagnosis date and histological type, were also obtained from the endoscopy centers for the cases. The reviewers gave enough time to the participants to think about each question and provide the most appropriate answer that best fits them. Especially about the nutrition issues, similar questions were asked in different wordings e.g. they were asked how frequently they consume hot beverages or if they mostly consume dark or light tea in different items to make intervariable consistency checks and minimize recall bias. Besides black tea, several kinds of infusion teas and soups were mentioned among the hot beverages consumed.

 Ethical approval for this study was obtained from Board of Ethical Committee of Herat University. Before starting the study, permission was also obtained from the local Public Health Administration of the Herat province. Furthermore, the objectives and procedure of the study were explained to every participant and a written consent was taken from people, who met the requirements of inclusion.

 Data was analyzed by the research team using IBM SPSS version 21 (IBM Corp. Released 2012. IBM SPSS Statistics for Windows, Version 21.0. Armonk, NY: IBM Corp.) at the Institute of Public Health, Hacettepe University, Ankara, Turkey. Descriptive statistics of the variables were presented as numbers (n) and percentages (%). The chi-square test was used to observe the difference between two categorical variables, while the Mann-Whitney U test was used for determining the difference between independent continuous variable without normal distribution. The findings were presented using marginal and contingency tables. In bivariate analysis, all independent variables with p-values less than 0.20 were entered into the model; logistic regression enter method was used for the strength of the association between dependent and independent variables. Odds ratios (ORs) and 95% confidence intervals (95% CI) were calculated and *P* values less than 0.05 were considered as statistically significant. Analyses were repeated solely for ESCCs, after performing them for all ECs. In univariate and multivariate (EC and SCC models) analyses, previous BMI was used as two categories (underweight and normal weight vs. overweight and obese); because of the lower number of subjects with underweight and obesity, underweight was combined with normal weight and obesity was combined with overweight.

## Results

 We presented the results of the study in two parts, for ECs and for ESCCs. The results of univariate analyses of EC and ESCC were presented jointly; however, the results of multivariate analyses were presented in two separate tables for EC and ESCC.

 In this study, 93 (51.7%) cases of EC were men and 87 (48.3%) cases were women while in the control group, the figures were 105 (55.6%) and 84 (44.4%) respectively; males and females were distributed equally between the case and control LR (*P*= 0.454). The median age of the EC cases was 56.3 which was not significantly different (*P*= 0.701) from the controls (56.0). Similarly, for ESCC cases and controls, the median age was 55.9 and 56 years, respectively, and the difference was not statistically significant (*P*> 0.05). The males accounted for 47.9% in ESCC cases and 55.6% in controls, while females accounted for 52.1% of ESCC cases and 44.4% of controls; a statistically significant difference was not observed between ESCC cases and control regarding sex (*P*= 0.167) (Table 1).

**Table 1:** Unadjusted Odds Ratios and 95% Confidence Intervals by potential risk factors for Esophageal Cancer, Herat Province Afghanistan 2017

**Characteristics**	**EC**			**ESCC**		
	**Cases** **No. (%)**	**Controls** **No. (%)**	**OR (95%CI)**	**Cases** **No. (%)**	**Controls** **No. (%)**	**OR (95% CI)**
Gender						
Female	87 (48.3)	84 (44.4)	Reference	76 (52.1)	84 (44.4)	Reference
Male	93 (51.7)	105 (55.6)	0.855 (0.567–1.288)	70 (47.9)	105 (55.6)	0.736 (0.478–1.136)
Literacy						
Literate	20 (11.1)	4 (22.8)	Reference	16 (11.0)	43 (22.8)	Reference
Illiterate	160 (88.9)	146 (77.2)	2.356 (1.325–4.191)	130 (89.0)	146 (77.2)	2.393 (1.286–4.451)
Marital Status						
Currently not married	21 (11.7)	20 (10.6)	Reference	15 (10.3)	20 (10.6)	Reference
Currently married	159 (88.3)	169 (89.4)	0.896 (0.468–1.716)	131 (89.7)	169 (89.4)	1.033 (0.509–2.096)
Ethnicity						
Pashtun	74 (41.1)	91 (48.1)	Reference	63 (43.2)	91 (48.1)	Reference
Tajik	91 (50.6)	77 (40.7)	1.453 (0.943–2.237)	72 (49.3)	77 (40.7)	1.350 (0.857–2.127)
Other	15 (8.3)	21 (11.1)	0.878 (0.423–1.823)	11 (7.5)	21 (11.1)	0.756 (0.340–1.678)
Residence						
Urban	36 (20.0)	63 (33.3)	Reference	28 (19.2)	63 (33.3)	Reference
Rural	144 (80.0)	126 (66.7)	2.000 (1.245–3.213)	118 (80.8)	126 (66.7)	2.107 (1.264–3.514)
Self-reported SES						
Good	78 (7843.3)	123 (65.1)	Reference	62 (42.5)	123 (65.1)	Reference
Bad	102 (56.7)	66 (34.9)	2.443 (1.601–3.712)	84 (57.5)	66 (34.9)	2.524 (1.627- 3.943)
Working Status						
No	101 (56.1)	111 (58.7)	Reference	86 (58.9)	111 (58.7)	Reference
Yes	79 (43.9)	78 (41.3)	1.113 (0.737 - 1.682)	60 (41.1)	78 (41.3)	0.993 (0.640 - 1.539)
Tobacco usage						
Never	91 (50.6)	95 (50.3)	Reference	74 (50.7)	95 (50.3)	Reference
Ever	89 (49.4)	94 (49.7)	0,988 (0.657 - 1.487)	72 (49.3)	94 (49.7)	0.983 (0.638 - 1.515)
Previous BMI						
Underweight + Normal	137 (76.1)	169 (89.4)	Reference	114 (78.1)	169 (89.4)	Reference
Overweight and Obese	43 (23.9)	20 (10.6)	0.377 (0.212–0.671)	32 (21.9)	20 (10.6)	0.644 (0.451–0.920)
Family history of Esophageal Ca						
No	154 (85.6)	181 (95.8)	Reference	121 (82.9)	181 (95.8)	Reference
Yes	26 (14.4)	8 (4.2)	3.819 (1.680–8.681)	25 (17.1)	8 (4.2)	4.675 (2.041- 10.707)

Chi-Square test; OR, Odds Ratio; EC, Esophageal cancer; ESCC, Esophageal squamous cell carcinoma.

 In the univariate analyses, focusing on the lifestyle characteristics of people in EC groups; literacy_illeterate_ (OR = 2.356, 95% CI = 1.325–4.191), residence_rural_ (OR = 2.000, 95% CI = 1.245–3.213), SES_bad_ (OR = 2.443, 95% CI = 1.601–3.712), previous BMI_overweight_ (OR = 0.377 (0.212–0.671) and positive family history of EC_yes_ (OR = 3.819, 95% CI = 1.680–8.681) were found to show statistically significant differences between case and control groups in EC (Table 1).

 We found significantly elevated risks for those who reside in rural areas (versus urban), are illiterate (versus literate) and have poor SES (versus good) regarding ESCC. Unadjusted ORs were 2.107 (95% CI = 1.264–3.514), 2.393 (95% CI = 1.286–4.451) and 2.524 (95% CI = 1.627–3.943) for ESCC in the univariate analyses (Table 1).

 When evaluating the nutritional contents, consumption of dairy products (milk, yogurt and cheese) was found to be significantly different between case and control groups for both EC and ESCC. Red meat, dried meat, salt, very hot beverage, a local food (*qulurtoroosh*) and dark tea consumption were also distributed significantly differently between cases and controls for both EC and ESCC (Table 2).

**Table 2:** Unadjusted Odds Ratios and 95% Confidence Intervals by potential risk factors of food consumption for Esophageal Cancer, Herat province Afghanistan 2017

**Characteristics**	**EC**			**ESCC**		
	**Cases** **No. (%)**	**Controls** **No. (%)**	**OR (95% CI)**	**Cases** **No. (%)**	**Controls** **No. (%)**	**OR (95% CI)**
Milk						
Never	55 (30.6)	76 (40.2)	Reference	47 (32.2)	76 (40.2)	Reference
Sometimes	77 (42.8)	94 (49.7)	1.131 (0.715 - 1.792)	59 (40.4)	94 (49.7)	1.015 (0.623 - 1.653)
Daily & Often	48 (26.7)	19 (10.1)	3.490 (1.850–6.584)	40 (27.4)	19 (10.1)	3.404 (1.766–6.561)
Yoghurt						
Never	33 (18.3)	27 (14.3)	Reference	31 (21.2)	27 (14.3)	Reference
Sometimes	80 (44.4)	112 (59.3)	0.584 (0.325–1.047)	63 (43.2)	112 (59.3)	0.489 (0.268–0.893)
Every meal + Daily + Often	67 (37.2)	50 (26.5)	1.096 (0.586–2.051)	52 (35.6)	50 (26.5)	0.905 (0.474–1.727)
Water						
Protect water	92 (51.1)	114 (60.3)	Reference	69 (47.3)	114 (60.3)	Reference
Non protect water	88 (48.9)	75 (39.7)	1.453 (0.962–2.197)	77 (52.7)	75 (39.7)	1.696 (1.096–2.624)
Cheese						
Never	140 (77.8)	175 (92.6)	Reference	116 (79.5)	175 (92.6)	Reference
Sometimes	18 (10.0)	10 (5.3)	2.250 (1.007–5.029)	14 (9.6)	10 (5.3)	2.112 (0.907–4.915)
Every meal + Daily + Often	22 (12.2)	4 (2.1)	6.875 (2.315–20.413)	16 (11.0)	4 (2.1)	6.034 (1.967- 18.504)
Red Meat						
Never	18 (10.0)	18 (9.5)	Reference	13 (8.9)	18 (9.5)	Reference
Sometimes	92 (51.1)	135 (71.4)	0.681 (0.337–1.379)	73 (50.0)	135 (71.4)	0.749 (0.347–1.613)
Daily & Often	70 (38.9)	36 (19.0)	1.944 (0.903–4.187	60 (41.1)	36 (19.0)	2.308 (1.012–5.263)
Dried Meat						
Never	34 (18.9)	30 (15.9)	Reference	28 (19.2)	30 (15.9)	Reference
Sometimes	79 (43.9)	140 (74.1)	0.497 (0.283–0.874)	62 (42.5)	140 (74.1)	0.474 (0.261–0.860)
Daily & Often	67 (37.2)	19 (10.1)	3.111 (1.533–6.312)	56 (38.4)	19 (10.1)	3.157 (1.519–6.567)
Salt addition at the table						
Not use	103 (57.2)	139 (73.5)	Reference	83 (56.8)	139 (73.5)	Reference
Use	77 (42.8)	50 (26.5)	2.078 (1.341–3.219)	63 (43.2)	50 (26.5)	2.110 (1.331–3.343)
Hot beverage						
Never	74 (41.1)	138 (73.0)	Reference	62 (45.6)	138 (73.0)	Reference
Every meal	106 (58.9)	51 (27.0)	3.876 (2.503 - 6.003	84 (54.4)	51 (27.0)	3.666 (2.316 - 5.803)
**Local food (Qulurtoroosh) only in winter**						
Never + Rarely	145 (80.6)	185 (97.9)	Reference	117 (80.1)	185 (97.9)	Reference
Daily& Often	35 (19.4)	4 (2.1)	11.160 (3.882–32.131)	29 (19.9)	4 (2.1)	11.464 (3.931–33.442)
**Fresh fruit**						
Never	21 (11.7)	16 (8.5)	Reference	19 (13.0)	16 (8.5)	Reference
Sometimes	94 (52.2)	88 (46.6)	0.813 (0.603 - 2.505)	77 (52.7)	88 (46.6)	0.737 (0.354–1.532)
Daily & Often	65 (36.1)	85 (45.0)	0.582 (0.282–1.204)	50 (34.2)	85 (45.0)	0.495 (0.234–1.049)
Vegetable						
Never	64 (35.6)	80 (43.0)	Reference	53 (36.3)	80 (43.0)	Reference
Sometimes	78 (43.3)	80 (43.0)	1.219 (0.774–1.917)	61 (41.8)	80 (43.0)	1.151 (0.711 - 1.862)
Daily & Often	38 (21.1)	26 (14.0)	1.827 1.005–3.320)	32 (21.9)	26 (14.0)	1.858 (0.996–3.464)
**Cereal food**						
Not use	20 (11.1)	15 (7.9)	Reference	19 (13.0)	15 (7.9)	Reference
Use	160 (88.9)	174 (92.1)	0.689 (0.341–1.393)	127 (87.0)	174 (92.1)	0.576 (0.282–1.177)
**Acidic food**						
Never	44 (24.4)	62 (32.8)	Reference	37 (25.3)	62 (32.8)	Reference
Sometimes	88 (48.9)	77 (40.7)	1.610 (0.983–2.635)	71 (48.6)	77 (40.7)	1.545 (0.919–2.597)
Daily & Often	48 (26.7)	50 (26.5)	1.353 (0.778–2.352)	38 (26.0)	50 (26.5)	1.273 (0.708–2.288)
Tea (dark)						
Not use	87 (48.3)	151 (79.9)	Reference	78 (53.4)	38 (20.1)	Reference
Use	93 (51.7)	38 (20.2)	4.248 (2.680 - 6.732)	68 (46.6)	151 (79.9)	4.558 (2.815 - 7.381)

OR, Odds ratio; CI, Confidence interval; EC, Esophageal cancer; ESCC, Esophageal squamous cell carcinoma.

 In terms of food items, milk consumption (often and daily versus never) yielded an OR of 3.490 (95% CI, 1.850–6.584) for EC and 3.404 (95% CI = 1.766–6.561) for ESCC on univariate analysis. Similarly, the unadjusted ORs for cheese consumption (often, daily and in every meal versus never) (6.875, 95% CI = 2.315–20.413), dried meat consumption (often and daily versus never) (3.111, 95% CI = 1.533–6.312), ever versus never consumption of salt (2.078, 95% CI = 1.341–3.219), hot beverage (every meal versus never) (3.876, 95% CI = 2.503–6.003), qulurtoroosh (often and daily versus never) (8.114, 95% CI = 2.694-24.433), often and daily versus never consumption of vegetables (1.827, 95% CI = 1.005–3.320) and ever versus never consumption of dark tea (4.248, 95% CI = 2.680–6.732) were significantly higher in EC (Table 2).

 Consumption of five food items were strongly associated with ESCC as the ORs were observed to be greater than 3.0 in univariate analyses: milk (OR = 3.404, 95% CI = 1.766–6.561), cheese (OR = 6.034 95% CI = 1.967–18.504), dried meat (OR = 3.157 95% CI = 1.519–6.567), the local food named qulurtoroosh (OR = 12.953, 95% CI = 4.386–38.254), hot beverages (OR = 3.666 95% CI = 2.316–5.803) and dark tea (OR = 4.558, 95% CI = 2.815–7.381).In addition, consumption of non-protected water versus protected water, red meat (often, daily and in every meal versus never) and salt addition at the table (often, every meal versus never) also significantly increase the risk for ESCC (Table 2).

 In multivariate analyses, all variables associated with EC in the univariate analyses were put in the model and backward elimination regression analysis was applied. Since “economic status” had collinearity with the variables “education level” and “place of residence”, only economic status was put into the model and the other two variables were left out of the multivariate analyses for both EC and ESCC. Although the age variable was not significant in univariate analyses, it was included in all models. Results of the adjusted final models are presented in Table 3 and Table 4.

**Table 3:** EC Data Variables in the Model

**Variables** **Exposure to**	***P*** **Value**	**OR**_adjusted_	**95% Confidence Interval**	
			**Lower**	**Upper**
Sex_male_	0.059	1.874	0.939	3.400
Age	0.219	0.984	0.960	1.009
Previous BMI_overweight_	< 0.001	0.112	0.049	0.257
Very hot beverage_yes_	< 0.001	3.482	1.920	6.312
Qulurtoroosh_yes_	< 0.001	9.424	2.762	32.155
Dark tea_yes_	< 0.001	3.016	1.667	5.460
Poor economic status_yes_	0.065	1.741	0.964	3.142
EC history among relatives_yes_	0.719	1.196	0.452	3.166
Dried meat_yes_	0.967	0.984	0.472	2.052
Vegetable_yes_	0.154	0.628	0.331	1.191
Fruit_yes_	0.428	1.528	0.524	4.459
Constant	0.233	0.258		

OR, odds ratio; EC, esophageal cancer. *P* < 0.05 statistically significance, subscripts indicate risk categories.

**Table 4:** ESCC Data Variables in the Models

**Variables** **Exposureto**	***P*** **Value**	**OR**_adjusted_	**95% Confidence Interval**	
			**Lower**	**Upper**
Sex_male_	0.038	2.268	1.238	4.153
Age	0.104	0.979	0.953	1.005
Previous BMI_overweight_	0.029	0.215	0.117	0.431
Very hot beverage_yes_	0.005	2.253	1.271	3.996
Qulurtoroosh_yes_	0.003	5.679	1.787	18.815
Dark tea_yes_	0.001	2.757	1.531	4.967
Poor economic status_yes_	0.049	1.783	1.007	3.177
EC history among relatives_yes_	0.007	4.161	1.465	11.815
Dried meat_yes_	0.406	1.493	0.581	3.836
Vegetable_yes_	0.150	0.640	0.349	1.174
Fruit_yes_	0.078	2.595	0.898	7.502
Constant	0.001	0.005		

OR, odds ratio; EC, esophageal cancer; BMI, body mass index. *P*< 0.05 statistically significance, subscripts indicate risk categories.

 The EC logistic regression (enter method) model included the following variables: sex, age, previous BMI, SES, EC history among relatives, consumption of dark tea, very hot beverage, consumption of fruit, consumption of vegetable, qulurtoroosh and dried meat; as the result of model, consumption of very hot beverage, consumption of qulurtoroosh, drinking dark tea and having previous lower BMI were evaluated in the model which indicated that these variables were independently associated with the increasing risk of EC (Table 3). Drinking very hot beverage was found to increase the risk significantly for EC by almost 3.5-folds (OR = 3.482, 95% CI = 1.920–6.312). The risk of EC was about 9.4 times higher among participants who consumed qulurtoroosh (OR = 9.424, 95% CI = 2.762–32.155). Dark tea consumption increased the risk of EC by almost three-folds (OR = 3.016, 95% CI = 1.677–5.460). However, having previous high BMI (overweight & obese) was found to be protective (OR = 0.112, 95% CI, 0.049–0.257).

 For multivariate logistic regression of ESCC; sex, age, previous BMI, EC history among relatives, dried meat, qulurtoroosh, very hot beverage, dark tea, consumption of fruit, consumption of vegetable and SES variables were analyzed in the enter model.

 The result of multivariate analysis for ESCC showed that sex, very hot beverage, qulurtoroosh, dark tea, previous BMI, SES and EC history among relatives were independently associated with the outcome variable. Result of the enter model are presented in Table 4.

 Being male was found to increase the risk of ESCC by 2.268 times (95% CI = 1.238–4.153). Four factors, consumption of very hot beverage, consumption of qulurtoroosh, drinking dark tea and EC history among relatives, were found to increase the risk of ESCC by almost 2-6 folds. The ORs for consumption of hot beverage, qulurtoroosh, dark tea, EC history among relatives and poor SES were found to be 2.253 (95% CI = 1.271–3.996), 5.679 (95% CI = 1.787–10.845), 2.757 (95% CI = 1.531–4.967), 4.161 (95% CI = 1.465–11.815) and 1.783 (95% CI = 1.001–3.177), respectively. Also, having previous high BMI decreased the risk significantly for ESCC by 0.215 (95% CI = 0.117–0.431) (Table 4).

## Discussion

 The result of our study indicate that consumption of very hot beverage was independently associated with EC and ESCC. Participants who consumed very hot beverages were 4 times more likely to develop EC as compared to participants who consumed normal or warm beverages. Furthermore, consumption of very hot beverages was also associated with a 4-time higher risk of developing ESCC compared to consumption of normal or warm beverages. Many studies including systematic reviews and meta-analyses have identified hot beverages as a risk factor for EC and ESCC. These findings are consistent with the results of our study. A plausible reason is that beverages with high temperature cause recurrent thermal injury to the esophagus and a significant increase up to 12-fold in the risk of EC.^11-13^ The International Agency for Research on Cancer (IARC) classified very hot beverages at above 65 degrees Celsius as probably carcinogenic for humans.^14-17^

 In line with our study, the association of hot beverage consumption and the risk of ESCC has been confirmed in many studies including a meta-analysis in which the authors indicated a twice higher risk of ESCC among hot beverage consumers compared to normal or warm beverage drinkers.^18,19^

 In our study, a local food called “qulurtoroosh”, a sour creamy food, was found to be a risk factor for EC which calls for attention. The risk of EC was almost 12 times higher and the risk of ESCC was 10 times higher among participants who consumed qulurtoroosh compared to participants who did not consume it (daily or never). Qulurtoroosh is made up with many types of seasonings such as black paper, sumac, ginger, and turmeric. These food seasonings are added to yoghurt, fresh ground tomato and flour to make a dough. The dough is then incubated at room temperature for 24–48 hours, after which it is dried and turned to powder. The powder is usually served as soup, and some people serve it with chopped pieces of bread and dried meat. The dried meat is normally kept at room temperature but covered with a lot of salt to prevent its spoiling. When dried meat is served with qulurtoroosh, the meat is not completely washed, so qulurtoroosh served with dried meat is very salty and fatty. To the best of our knowledge, there is no study mentioning this local food; however, our finding with regards this food might be attributed to the ingredients contained in the food such as dried meat and very high salt levels. A previous study had shown a positive association between salt and esophageal carcinoma in Eastern Anatolia.^20^ In another study conducted in China, it was discovered that salted meat intake was associated with an increased risk of ESCC, showing an exposure–response relationship. The study further revealed that consumption of 50 g salted meat per week was related to an 18% increased risk of ESCC.^21^ Cross et al^22^ found a positive association between red meat intake and ESCC; also, Genkinger and Koushik^23^ concluded that red and processed meat intake was positively associated with the risk of esophagus cancer and other cancers.

 In our study, the association between dried meat and EC as well as ESCC was statistically significant in the bivariate analysis; however in the multivariate analysis, the significance of association with dried meat was lost in both EC and ESCC models. The reason that dried meat was neither associated with EC nor with ESCC independently might be the usage of dried meat together with qulurtoroosh in this study. Although one of the main ingredients of qulurtoroosh was dried meat, we included dried meat and qulurtoroosh separately in the models to investigate the association of both dried meat and qulurtoroosh separately. We think that the stronger observed association of qulurtoroosh with the EC and ESCC might have concealed the significance of dried meat in both models.

 Apart from the consumption of hot beverages, we found that consuming dark tea also increased the risk of both EC and ESCC; the risk of EC was 3 times and the risk of ESCC was 3 times higher among people who consumed dark tea compared to those who consumed light tea. Some artificial colors and other materials added to the tea without any kind of safety and quality control in the study area might be responsible for the findings about dark tea.^24^

 The study conducted in the Caspian Littoral of Iran in 1979, and the study by Islami et al conducted in high-risk areas in 2009, indicated that low socioeconomic status has strong association with the risk of EC. The authors mentioned that low or no intake of fresh fruit and vegetable, living in overcrowded and closed area, having less access to health services and screening programs and other unhealthy behaviors which are commonly associated with low socioeconomic status might influence EC.^25,26^ We found that participants with poor SES had a significantly higher risk for EC and ESCC.

 The findings of our study illustrated that obesity was negatively associated with EC and ESCC risk. In participants with underweight and normal weight, the risk of EC was 8 times and the risk of ESCC almost 9 times higher than the participants with higher body weight. There are many previous studies which support the result of our study for EC as well as ESCC. A study conducted in China found a strong inverse association between BMI and death from EC, with each 5 kg/m^2^ higher BMI associated with 25% lower EC mortality.^11^ Sankaranarayanan et al conducted a study in the Huai’an District of Jiangsu Province, China; they also found out that higher BMI was inversely correlated with EC, which supported the result shown by our study.^12^ A population-based case-control study of esophageal and gastric cancers conducted in Connecticut, New Jersey, and Western Washington, reported a tendency towards a decreasing risk of ESCC with increasing BMI.^13^ Furthermore, a cohort study conducted in Norway observed an inverse relationship between BMI and ESCC.^27^ Also, Lindkvist et al confirmed the inverse association of BMI with ESCC through a follow-up study. The Metabolic syndrome and Cancer project (Me-Can) which consisted of seven prospective cohorts in Austria, Norway and Sweden, showed the risk of ESCC as almost 3 times lower among participant with higher BMI than participants with normal BMI.^28^

 Human ESCC is one of the most aggressive malignancies, and most epidemiological studies have found prominent gender differences in the prevalence of ESCC. The age-standardized rate of ESCC for males in the less developed countries is 11.8/100 000 while it is 4.7/100 000 for females (the male: female incidence ratio is 2.4:1).^3^ Although ESCC is similarly distributed among males and females in the high risk areas of Iran and China,^12^ the study by Okello et al observed that compared to females, males are exposed to an almost 4-time higher risk of ESCC.^29^ In our study, we found that female sex was protective against ESCC, as females were exposed to half the risk of ESCC compared to males, which is consistent with the previous studies. Besides, considering that we adjusted the case and control groups for age and sex, we can speculate that because of the selection process, this difference might have been found to be attenuated in our study, and the OR for males compared to females might be much stronger in the real settings.

 Regarding the limitations of this study, we included only historically confirmed cases in this study; thus, participants with poor SES might not have adequate access to pathological examination due to their financial restrictions and this might be a source of bias. Self-reported SES and previous BMI might also to some extent be a cause of bias in our study. Due to the case-control design of this study, a direct causal inference of risk factors to EC could not be identified. Recall bias and sampling bias may also have been present. In addition, the fact that opium consumption, which is common in Afghanistan and defined as a risk factor for EC, was not evaluated in our study, is another limitation.

 In conclusion, this is the first study conducted to identify the risk factors for EC which is a prominent health problem in the region. We found that consuming very hot beverages, dark tea and a local food called qulurtoroosh are important risk factors for EC. Our findings will aid further studies and help in the implementation of protective measures for EC which is one of the leading types of cancers in the region.
